# Reactor environment during the Fukushima nuclear accident inferred from radiocaesium-bearing microparticles

**DOI:** 10.1038/s41598-020-58464-y

**Published:** 2020-01-28

**Authors:** Taiga Okumura, Noriko Yamaguchi, Hiroki Suga, Yoshio Takahashi, Hiroyo Segawa, Toshihiro Kogure

**Affiliations:** 1 The University of Tokyo, Department of Earth and Planetary Science, Graduate School of Science, 7-3-1 Hongo, Bunkyo-ku, Tokyo 113-0033 Japan; 2 Institute for Agro-Environmental Sciences, NARO, 3-1-3 Kannondai, Tsukuba, Ibaraki 305-0864 Japan; 3 National Institute for Materials Science, 1-1 Namiki, Tsukuba, Ibaraki 305-0044 Japan

**Keywords:** Nuclear chemistry, Materials science

## Abstract

Radiocaesium-bearing microparticles (CsMPs), which are substantially silicate glass, were formed inside the damaged reactor and released to the environment by the Fukushima Dai-ichi Nuclear Power Plant accident in March 2011. The present study reports several valuable findings regarding their composition and structure using advanced microanalytical techniques. X-ray absorption near-edge structure of Fe L_3_-absorption indicated that the oxidation state of the iron dissolved in the glass matrix of the CsMPs was originally nearly divalent, suggesting that the atmosphere in which the CsMPs were formed during the accident was considerably reductive. Another major finding is that sodium, which has not been recognised as a constituent element of CsMPs thus far, is among the major elements in the glass matrix. The atomic percent of Na is higher than that of other alkali elements such as K and Cs. Furthermore, halite (NaCl) was found as an inclusion inside a CsMP. The existence of Na in CsMPs infers that seawater injected for cooling might reach the inside of the reactor before or during the formation of the CsMPs. These results are valuable to infer the environment inside the reactor during the accident and the debris materials to be removed during the decommissioning processes.

## Introduction

A significant amount of radionuclides, including radiocaesium (^134^Cs and ^137^Cs), were released into the environment by the Fukushima Dai-ichi Nuclear Power Plant (FDNPP) accident in March 2011^1^. Radiation contamination around the nuclear plant is now mainly caused by ^137^Cs because of its relatively high amount and long half-life (30.2 years). Most of the released radiocaesium was in a gaseous state at the time of the accident, fell onto the ground, and fixed to mineral grains, such as partially vermiculitised biotite, on the ground^2^. However, part of the radiocaesium was incorporated into micron-sized particles inside the reactor and these particles, termed radiocaesium-bearing microparticles (CsMPs), were emitted from the damaged reactor. In addition to the CsMPs which are also termed Type-A particles in some literature, Type-B particles larger than several dozen microns were found around the nuclear plant^3–5^. Although Type-B particles generally possess higher radioactivity than Type-A particles, specific radioactivity of Type-A is far higher than that of Type-B. Based on the activity ratios of the radiocaesium isotopes (^134^Cs/^137^Cs), Type-A and B particles are considered to have been formed in the Unit 2 or 3 and Unit 1 Reactors, respectively, because the activity ratios are varied by fuel burnup differences among the units^6^. All the CsMPs analysed in this study are classified to Type-A particles because they were spherules of a few microns and caesium could be detected using X-ray composition analysis owing to the high specific radioactivity^4^.

CsMPs were first identified in aerosol filters collected in Tsukuba, Japan^7^. Microscopic analyses using synchrotron radiation (SR) X-ray and transmission electron microscopy (TEM) showed that the CsMPs are substantially silicate (SiO_2_) glass with Cl, K, Fe, Zn, Rb, Sn, and Cs dissolved in the glass as major components^8–10^. Some of these elements often show inhomogeneous distributions inside the CsMPs. In many CsMPs, the Cs concentration increases from the centre to the surface of the particles, suggesting that gaseous Cs was present in the reactor and diffused into the molten silicate particles following their formation^10,11^. When Cs is abundant near the surface, other alkali elements (K and Rb) are inclined to possess reversed distributions. Fe and Zn are also enriched near the surface in some CsMPs^11^. Results of quantitative analysis of the glass considering the radial distributions of the elements showed the SiO_2_ component reaches 60–70 wt%^10^. Further analysis using electron energy-loss spectroscopy (EELS) indicated that CsMPs do not contain boron^11^, which is easily incorporated into silicate glass if it exists in the surrounding atmosphere^12^. This infers that the B_4_C control rods created a eutectic alloy with stainless steel without vaporization during the accident. In this manner, characterization of CsMPs can provide information regarding the conditions inside the damaged reactors. However, a few issues relating to the composition of CsMPs remain inconclusive.

Among them is the valence state of transition metals such as iron in the CsMPs, which may reflect the redox state of the atmosphere in the reactor during the accident. X-ray absorption near-edge structure (XANES) analysis using an SR X-ray microbeam indicated the high oxidation states of Fe, Zn, Mo, and Sn in the CsMPs, i.e., Fe^3+^, Zn^2+^, Mo^6+^, and Sn^4+^, suggesting that the CsMPs were formed in an oxidative atmosphere^8^. However, inclusions of chromian magnetite (Fe^2+^(Cr^3+^, Fe^3+^)_2_O_4_) were often found in the CsMPs, which conversely suggests that the atmosphere was rather reductive^11^. This discrepancy should be solved to determine the atmosphere inside the damaged reactor which might have filled with hydrogen and superheated steam during the accident^13^.

Another issue is comprehensive determination of the chemical composition of the CsMPs. As previously mentioned, the absence of boron was confirmed by EELS in TEM but the existence of other light elements remains uncertain. Sodium is among the elements to be clarified. Although Cl in CsMPs was suggested to have originated in seawater injected into the reactor during the accident for cooling, the existence of Na has not been reported thus far, especially for type-A particles^9,10,14^. This leads a speculation that the seawater was not the origin of the Cl and did not sufficiently reach the inside of the reactor when the CsMPs were formed^15^. X-ray fluorescence analysis generally has low sensitivity for detection of light elements such as Na. Conventional energy-dispersive X-ray spectrometer (EDS) equipped to electron microscopes also cannot clearly confirm the presence of Na in CsMPs if Zn exists in samples because of the peak overlapping of Na Kα and Zn L (Na Kα: 1.041 keV, Zn Lα: 1.012 keV, Zn Lβ: 1.034 keV). Some previous research reported the Na peak in EDS spectra, however, the overlapping peaks of Na and Zn were blindly assigned to the Na peak in the papers. Although a wavelength-dispersive X-ray spectrometer, commonly equipped to an electron probe microanalyser, is able to resolve these peaks, Na in glass materials rapidly migrates and disappears because of intense electron-beam irradiation^16^. For these reasons, the presence of Na in CsMPs has not been clarified thus far.

In this study, we attempted to clarify these two issues using XANES with scanning transmission X-ray microscopy (STXM) and EDS with scanning TEM (STEM) paying careful attention to X-ray and electron-beam irradiation effects. The following new results with respect to the composition and structure of CsMPs leads to valuable insights into the condition of the reactor during the accident.

## Results

### Oxidation state of iron in the CsMPs

**Figure 1:**
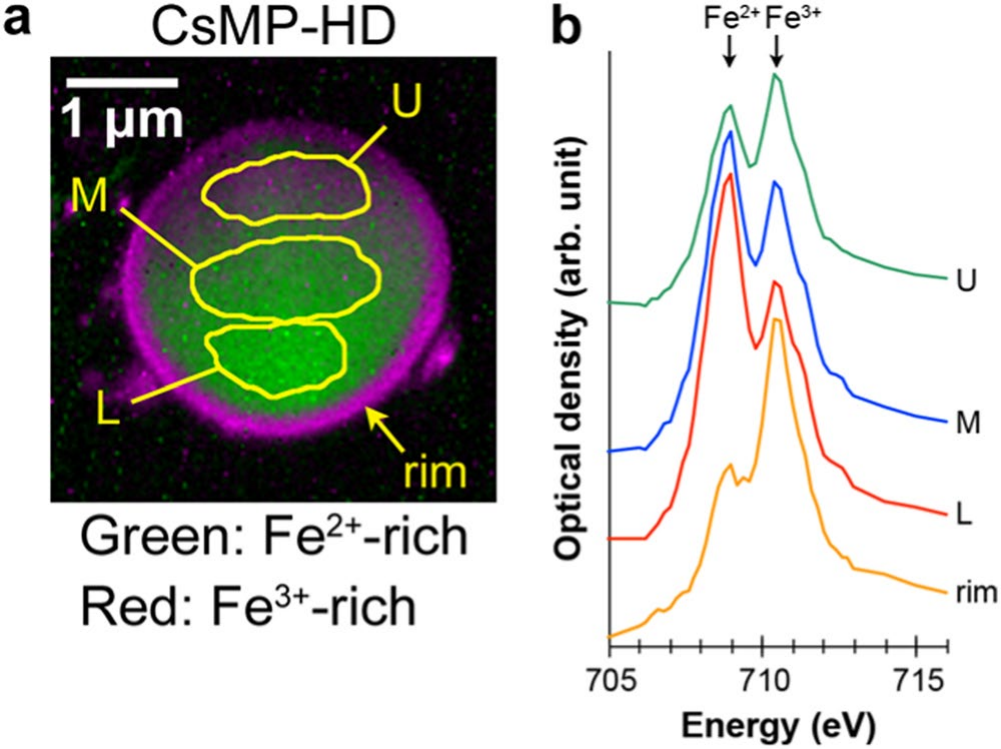
Oxidation state of iron in CsMP-HD determined using STXM (step size: 30 nm; dwell time: 5 ms). (**a**) Fe oxidation state map. (**b**) XANES spectra acquired from the areas indicated by L, M, and U in. (**a**) The spectrum from the rim region is also shown.

**Figure 2:**
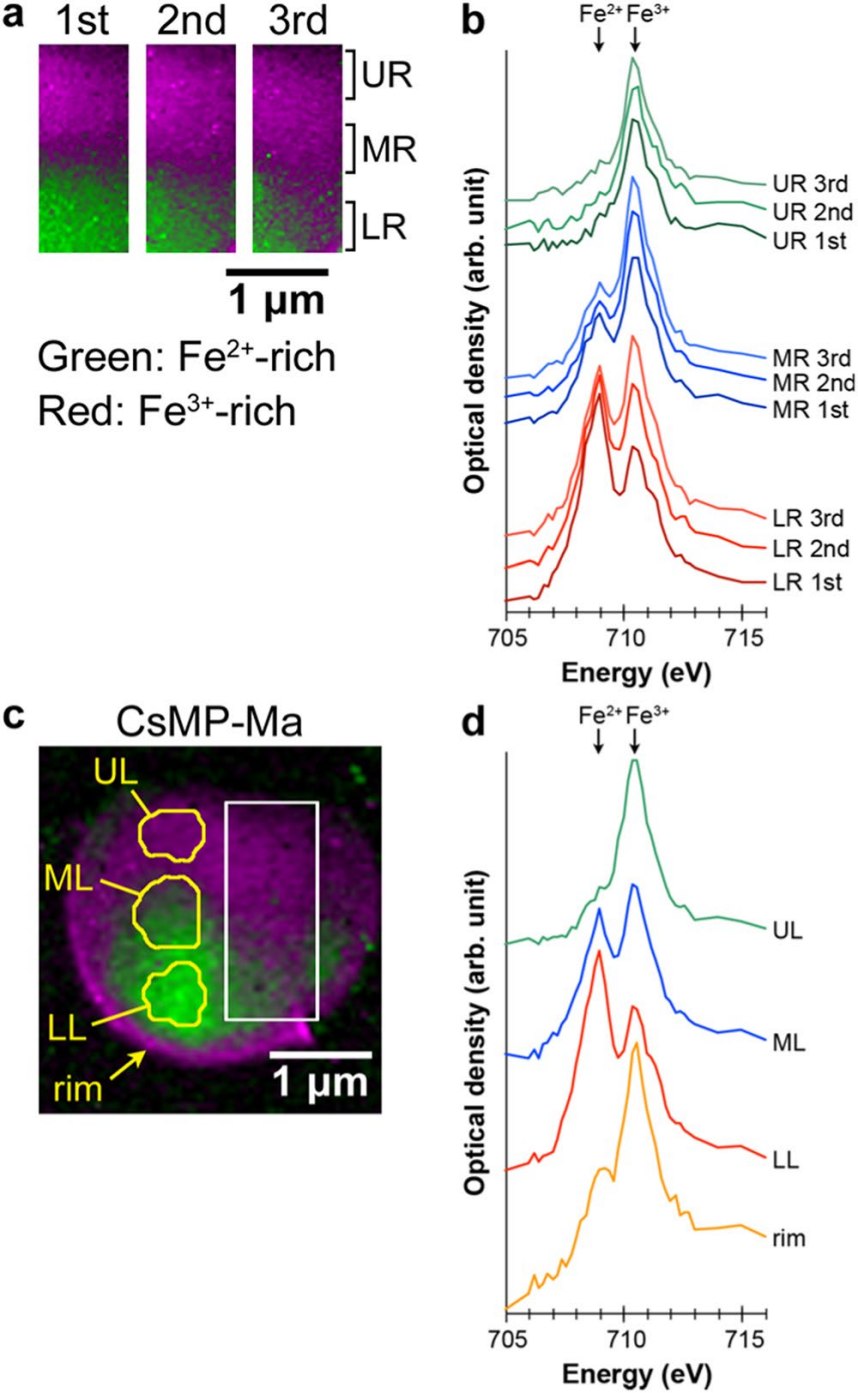
Oxidation state of iron in CsMP-Ma determined using STXM. (**a**) Fe oxidation state maps of the repeatedly measured area indicated by the rectangle in. (**c**) The area was measured three times (step size: 30 nm; dwell time: 5 ms). (**b**) XANES spectra acquired from the areas indicated by LR, MR, and UR in. (**a**) (**c**) Fe oxidation state map of the entire area of CsMP-Ma (step size: 50 nm; dwell time: 5 ms). (**d**) XANES spectra acquired from the areas indicated by LL, ML, and UL in. (**c**) The spectrum from the rim region is also shown.

To determine the oxidation state of iron, which is sensitive to the redox state of surrounding atmosphere, XANES analysis for the Fe L_3_ edge was conducted using STXM. In the Fe L_3_ edge, the peak energy of Fe^2+^ (~708.2 eV) is lower than that of Fe^3+^ (~709.6 eV) and their ratio can be estimated from the intensity ratio of the separated peaks^17^. For STXM measurement, thin specimens of two CsMPs, termed CsMP-HD and CsMP-Ma, were prepared for penetration by low-energy X-ray using a focused ion beam (FIB) apparatus. Figure 1a shows the Fe oxidation state map of CsMP-HD. In the map, divalent and trivalent iron are expressed by the gradation of green and red colours, respectively, using a singular value decomposition (SVD) method based on the spectra acquired from Fe^2+^- and Fe^3+^-rich regions^18^. In this study, the peak energy at 709 eV (Fe^2+^) and 710.4 eV (Fe^3+^) was slightly higher than the reference values because of inaccurate initial energy calibration. Averaged XANES spectra for the Fe L_3_ edge from the specific regions are shown in Fig. 1b. The map in Fig. 1a clearly shows that the distribution of the iron oxidation state is inhomogeneous inside the CsMP. In the vicinity of the periphery of ~0.2 μm in thickness, the iron is completely oxidised to Fe^3+^. Inside the CsMPs, Fe^2+^ is dominant but Fe^3+^ increases toward the upper region. The thin specimens for STXM were prepared using FIB and the sample thinned toward the upper region, as confirmed by the relative thickness measurement using EELS (the detail of the measurement is described in the next section). This implies that the surfaces of the thin specimens were more oxidised than the inside, which is definitely an artefact and probably formed during sample preparation using FIB or XANES measurement using STXM. To evaluate the X-ray irradiation effect, a specific part of CsMP-Ma was repeatedly measured three times using STXM (Fig. 2a,b). The red-coloured area corresponding to Fe^3+^ expanded and the Fe^3+^ peak at 710.4 eV increased with repetitive measurement, indicating that the specimen was progressively oxidised from its surface because of X-ray irradiation. The STXM analysis for the whole area of CsMP-Ma following the repetitive measurement also showed a comparable result to that shown in Fig. 1 (Fig. 2c), but the XANES spectra acquired from the specific regions inside the CsMPs indicated that the Fe^3+^ peaks in CsMP-Ma were higher than those in CsMP-HD (Fig. 2d). This result is because CsMP-Ma was thinner than CsMP-HD as confirmed again by the relative thickness measurement using EELS. Considering the preliminary X-ray irradiation for searching the analysis area, it is likely that the state of iron in the CsMPs before the measurement was nearly divalent except in the vicinity of the rim of the CsMPs. To evaluate the effects of FIB on the oxidation state, the synthetic glass with a similar composition was thinned in the same manner as CsMPs and analysed using STXM (Fig. S1). The means to synthesise the glass is described in the method section and its composition was determined using inductively coupled plasma (ICP) analysis as listed in Table S1. This glass was synthesised by using the previously reported composition of CsMPs as a reference, although some points are different from the actual CsMPs (for instance, Cl is not included in this glass)^10^. According to the XANES spectrum of the synthetic glass, the ratio of Fe:^2+^Fe^3+^ is approximately 2:3. On the other hand, Mössbauer spectroscopy for the bulk synthetic glass also showed that the ratio is approximately 2:3 (Fig. S2 and Table S2). Thus, sample preparation by FIB had little effects on the oxidation state of iron in glassy materials.

**Figure 3:**
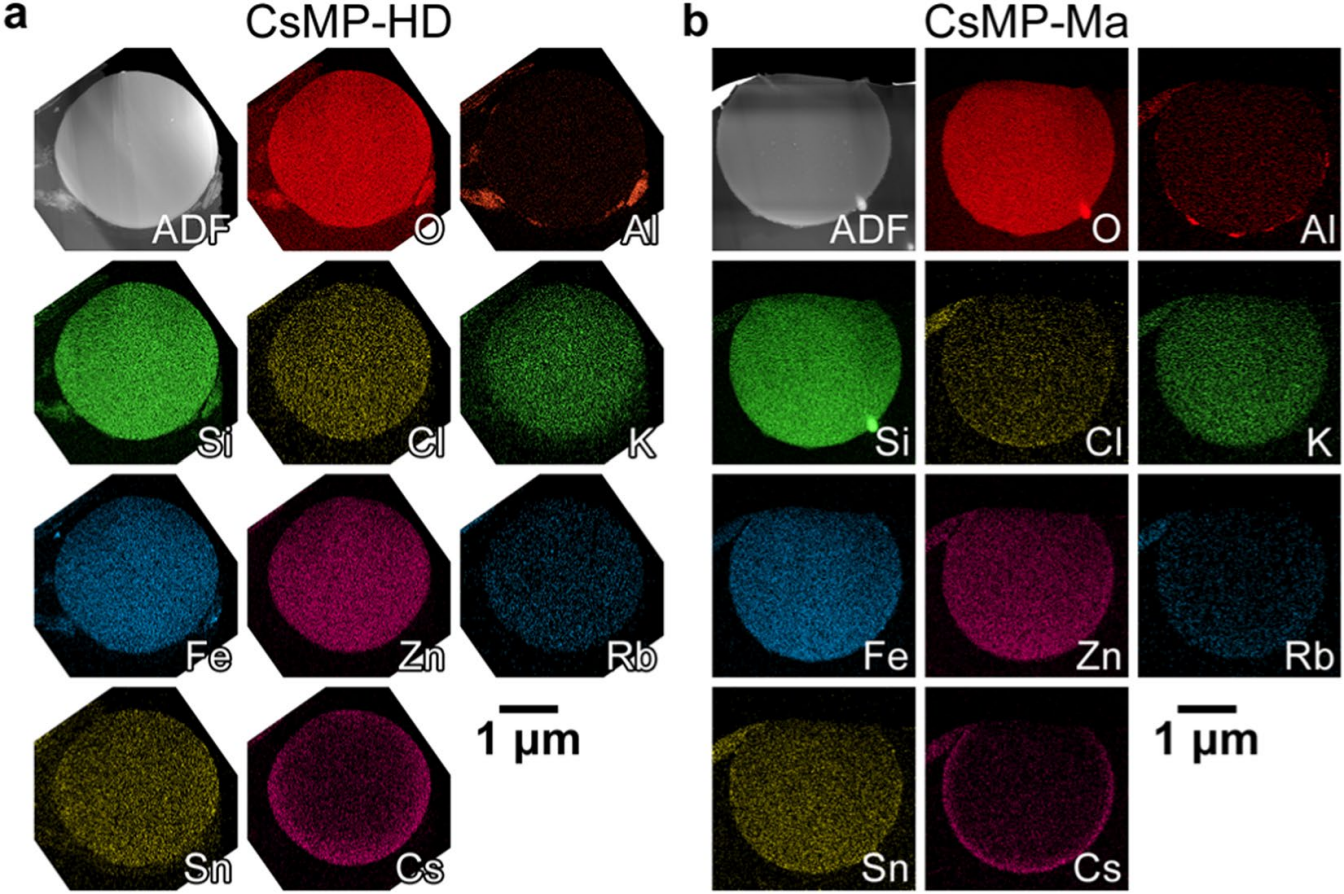
ADF images and corresponding elemental maps of CsMP-HD (**a**) and CsMP-Ma (**b**) obtained using STEM-EDS.

Following STXM analysis, elemental mapping was conducted using STEM-EDS for CsMP-HD and CsMP-Ma (Fig. 3). Their constituent elements were consistent with those of previously reported CsMPs. The Cs concentration increased from the centre to the surface, and in particular, Cs was predominantly concentrated at the rim region in CsMP-Ma. In contrast, K had a distribution opposite to that of Cs, which has also been previously reported. However, transition metals (Fe, Zn, and Sn) are homogeneously distributed in these CsMPs. It is probable the distributions of these elements and that of the iron valence state are not affected by each other.

### Sodium in the CsMPs

**Figure 4:**
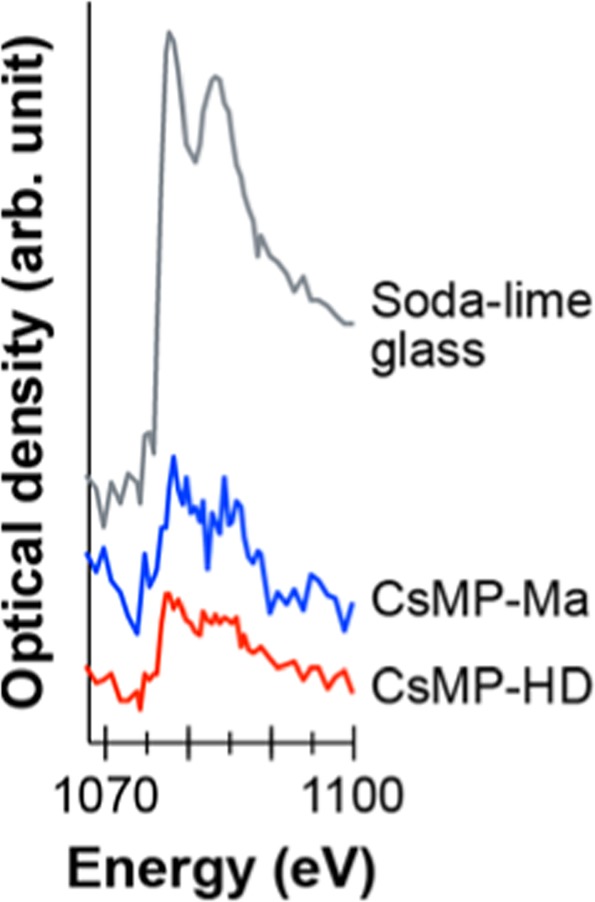
XANES spectra of commercial soda-lime glass, CsMP-HD, and CsMP-Ma at the Na K edge acquired using STXM (step size: 50 and 100 nm for CsMP-HD and CsMP-Ma, respectively; dwell time: 5 ms).

To examine the presence of Na in the CsMPs, Na K edge XANES spectra were acquired using STXM for the same thin specimens of CsMP-HD and CsMP-Ma (Fig. 4). A commercial soda-lime glass containing 13.7 wt.% Na_2_O was also measured as a reference of Na-containing glass and showed two peaks at the Na K edge^19^. The spectra from CsMP-HD and CsMP-Ma clearly showed an edge jump with two peaks at the Na K edge, indicating the presence of Na in the CsMPs, although the Na content is significantly lower than that of the soda-lime glass based on their peak heights.

**Figure 5:**
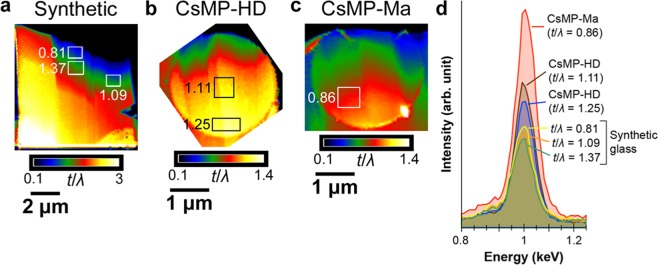
Relative thickness maps of synthetic glass (**a**), CsMP-HD (**b**), and CsMP-Ma. (**c**) Rectangles show the location where the EDS spectra were obtained. The values next to the rectangles indicate the relative thickness of each rectangular area. (**d**) EDS spectra obtained from the rectangles in (**a–c**) at the Na Kα and Zn L peaks.

Based on this result, EDS analysis must also detect an Na Kα signal in spite of its peak overlapping with Zn L. Therefore, we carefully compared EDS spectra from the CsMPs to those from the synthetic glass with a similar composition but without Na. Before the comparison of EDS spectra, the sample thickness was estimated using EELS. Thickness *t* of TEM samples can be evaluated using the equation *t*/*λ* = Ln(*I*_T_/*I*_0_), where *λ* is the inelastic mean free path of electrons with specific incident energy in the specimen, *I*_T_ is the total transmitted electron intensity, and *I*_0_ is the zero-loss peak intensity^20^. Assuming the values of *λ* are comparable between the synthetic glass and the investigated CsMPs, the relative thickness *t*/*λ* can be used for thickness evaluation. Figure 5a–c shows the determined relative thickness maps for the synthetic glass, CsMP-HD, and CsMP-Ma.

Next, the EDS spectra were acquired from the rectangular areas shown in Fig. 5a–c and normalised by each Zn Kα peak intensity. The overlapping peaks of Zn L and Na Kα in the normalised spectra are shown in Fig. 5d. Because the synthetic glass did not contain Na, their peaks arose only from Zn L. The peak intensity of the synthetic glass decreased with increasing thickness because of X-ray absorption inside the samples. The peaks from CsMP-HD and CsMP-Ma are obviously higher than those from the synthetic glass although their sample thickness was similar. The difference in the peak intensity originated from the Na Kα, indicating the presence of Na in the CsMPs.

Furthermore, the Na/Si ratio in the CsMPs was estimated by accurately calculating the intensity of the Na Kα and Si Kα peaks for the EDS spectra acquired from the rectangular areas shown in Fig. 5a–c. Because the energy of the X-ray of Na Kα and Si Kα is rather low, correction for the X-ray absorption inside the specimen is necessary to determine the accurate intensity of their peaks. The detailed calculation for the absorption correction is described in the Supplementary Information. As a result, the centre and periphery of CsMP-HD and CsMP-Ma showed an Na/Si atomic ratio of 0.185, 0.132, and 0.172, respectively (Table 1). The atomic ratio between other alkali elements (K, Rb, and Cs) and Si calculated using the Cliff-Lorimer method is also shown. The data clearly indicates that CsMPs contain much more Na than other alkali elements in the glass matrix, even in the periphery of CsMP-HD where the Cs concentration is higher. Furthermore, the Na/Si ratio in other three CsMPs was also calculated in the same manner (Fig. S4 and Table 1). First is termed CsMP-Fc which is also described in the next section, second is CsMP-Sg which is a new specimen with homogeneous distribution of Cs (Fig. S5), and third is WHT-1 which were a previously reported CsMP with high concentration of Cs at the periphery^11^. Consequently, these CsMPs also contain more Na than other alkali elements although the Na/Si ratio in CsMP-Sg is relatively lower.

**Table 1:** Relative thickness (*t*/*λ*) and atomic ratio of the alkali elements and Si for CsMP-HD and CsMP-Ma.

	CsMP-HD (centre)	CsMP-HD (periphery)	CsMP-Ma	CsMP-Fc	CsMP-Sg	WHT-1 (centre)	WHT-1 (periphery)
*t*/*λ*	1.11	1.25	0.86	1.89	0.94	2.02	2.04
Na/Si	0.185 (3)	0.132 (2)	0.172 (3)	0.122 (1)	0.069 (1)	0.153 (3)	0.139 (6)
K/Si	0.055 (3)	0.041 (3)	0.037 (2)	0.018 (2)	0.027 (2)	0.041 (3)	0.024 (5)
Rb/Si	0.017 (2)	0.017 (2)	0.007 (1)	0.013 (1)	0.012 (1)	0.007 (2)	0.012 (4)
Cs/Si	0.013 (1)	0.039 (2)	0.006 (1)	0.051 (1)	0.063 (1)	0.007 (1)	0.053 (5)

### Halite included inside the CsMPs

**Figure 6:**
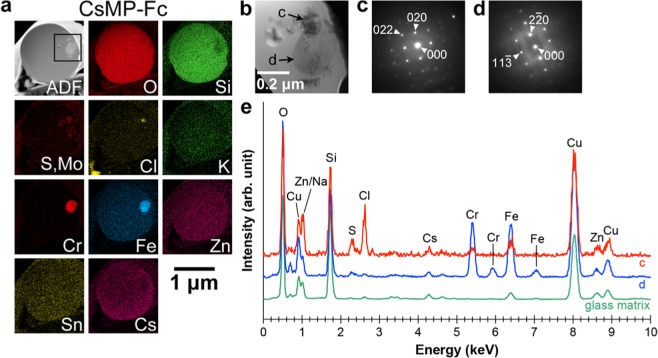
(S)TEM observation of CsMP-Fc. (**a**) ADF image and corresponding elemental maps. (**b**) Bright-field TEM image of the rectangular area in the ADF image in (**a**). (**c**) ED pattern obtained from the inclusion indicated by arrow c in (**b**), corresponding to halite observed along <100>. (**d**) ED pattern obtained from the inclusion indicated by arrow d in (**b**), corresponding to a spinel structure observed along <332>. (**e**) EDS spectra acquired from the inclusions indicated by arrows c and d in (**b**) and from the glass matrix. Cu peaks are from a grid supporting the specimen.

Another CsMP, CsMP-Fc, was observed using (S)TEM (Fig. 6). Three types of inclusions were identified in the elemental maps of the CsMP (Fig. 6a). The first inclusion indicated by arrow c in Fig. 6b was rich in Cl. Figure 6c shows the electron diffraction (ED) pattern acquired from the inclusion, which corresponded to halite (NaCl) observed along <100>. The second inclusion indicated by arrow d in Fig. 6b was rich in Cr and Fe. Its ED pattern shown in Fig. 6d was explained by a spinel structure observed along <332>. The third inclusion was rich in S and/or Mo and was presumed to be molybdenite (MoS_2_) which is also present in another CsMP^11^, although we failed to acquire its ED pattern. The EDS spectra of inclusions c and d and the glass matrix are shown in Fig. 6e. Inclusion c contained a large amount of Cl, and furthermore, the overlapping peak of Zn L and Na Kα was clearly higher than that of the glass matrix, indicating that the Na content was also high in the inclusion. This composition rich in Na and Cl also suggests that the inclusion is halite. The composition and ED pattern identified inclusion d as chromian spinel, which is a frequently observed inclusion inside CsMPs^11^. Thus, Na was present not only as a dissolved element in the glass matrix but also as a halite inclusion in CsMP-Fc.

## Discussion

The previous XANES analysis using an SR X-ray microbeam reported that Fe, Zn, Mo, and Sn in the CsMPs were present in high oxidation states as Fe^3+^, Zn^2+^, Mo^6+^, and Sn^4+^, respectively^8^. In this study, we obtained a different result based on the STXM-based XANES analysis that iron in the CsMPs is nearly divalent. The result in previous XANES research^8^ might reflect the properties of oxidised CsMPs because the iron in the CsMPs was rapidly oxidised resulting from X-ray irradiation during the experiments. Generally, the valence states of redox-sensitive elements including iron in glass are not strictly fixed and can change with X-ray irradiation^21–23^. In fact, iron oxidation was also observed depending on X-ray irradiation in our STXM measurements in spite of the sample chamber being purged with 0.1 atm of helium. Accordingly, iron in the CsMPs must be immediately oxidised when irradiated by X-ray in an ambient atmosphere. The presence of only divalent iron can constrain the oxygen fugacity of the reactor atmosphere during CsMP formation from a thermodynamics perspective^24^. Nevertheless, it may be possible that the CsMPs analysed in the present study have different properties from those reported in the previous research, because the numbers of CsMPs measured in this and previous studies are limited. STXM analysis of iron also showed that the rim of both CsMPs consists of trivalent iron regardless of the X-ray irradiation. One possible explanation is that iron in the rim regions was oxidised in the environment. Because CsMPs with cassiterite (SnO_2_) precipitation were previously reported^10,25^, the surface condition of CsMPs can be modified in the environment after release from the reactor. Another possible explanation is that the reactor atmosphere changed from reductive to oxidative during CsMP formation. We previously reported CsMPs with a higher concentration of Fe and Zn near the surface^11^ and such an inhomogeneous distribution can also occur via atmospheric change because divalent cations in silicate glass tend to diffuse outward when the glass is exposed to an oxidative atmosphere after glass formation in a reductive atmosphere^26^. To explain the presence of trivalent iron at the rim regions, more CsMPs should be examined to confirm whether it is a general feature of CsMPs.

Both STXM and EDS analyses confirmed that Na is among the major constituent elements in the CsMPs. The possible origin of the Na is presumed to be seawater injected for cooling during the accident. In this case, it is certain that the seawater was heated and concentrated until sea salt precipitation occurred because halite was found inside the CsMPs. But it is still possible that Na may have originated from other substances such as structural materials. The existence of Na in CsMPs can also explain the reason why Fe and Zn were not precipitated as a separate phase but were dissolved in the glass matrix. It is known that an immiscibility gap exists in binary RO-SiO_2_ systems in silicate melt, but a small amount of alkali elements can eliminate the immiscibility^27,28^. Although the glass matrix of CsMPs contains a certain amount of K and Cs as alkali elements, many CsMPs show a lower alkali concentration near the centre^10^. The synthetic glass which simulated the composition of CsMPs was segregated into (Si and alkali)-rich and (Fe and Zn)-rich phases in a previous study^10^ but this phase separation can be prevented by adding Na to the starting materials. Furthermore, the iron in the glass synthesised at an ambient atmosphere was mainly trivalent and the immiscibility is larger for trivalent compared to divalent iron^27^. Therefore, the glass synthesis under a controlled redox atmosphere and containing divalent iron also leads to elimination of immiscibility. If the glass which is more similar to CsMPs can be synthesised based on new information obtained in this study, such a synthetic glass can be used to infer the various properties of CsMPs. For instance, thermal and dissolution behaviour of CsMPs, which have been investigated by laboriously collecting actual CsMPs^29–31^, can be more easily and accurately elucidated. Furthermore, the atmosphere inside the damaged reactors during the accident can be estimated by determining the conditions for synthesising the same glass as CsMPs, providing useful information on the debris materials to be removed during decommissioning processes.

## Methods

### Collection of the CsMPs

CsMPs were collected from non-woven fabric cloth laid on a vegetable field approximately 25 km west-northwest from the FDNPP, “Nakadori” area in Fukushima Prefecture. The cloth was left outside for approximately 6 months following the accident. The cloth was then cut into fragments 15 × 15 mm in size and exposed to an imaging plate (BAS-2500, Fujifilm) for 10 min to confirm the presence of CsMPs as bright spots in the readout images. The isolation of individual CsMPs was conducted using a previously described method^32^. The CsMPs attached to the cloth were transferred to 10 mL of ion-exchanged water via ultrasonication. After removing the cloth from the water, the water was divided into 0.5-mL aliquots and the aliquots of water containing CsMPs were identified by measuring the radioactivity using an automatic gamma counter (Wizard2480, PerkinElmer). Ten millilitres of ion-exchanged water were added to each selected aliquot of water and these aliquots were again divided into 20 aliquots of 0.5 mL. The aliquots containing CsMPs were identified again. This process was repeated several times to isolate individual CsMPs from other unrelated particles. The water suspending the CsMPs was dropped onto a piece of double-sided Kapton tape (P-224, Nitto) and dried at ambient temperature. The tape was carbon coated and the CsMPs were searched using a scanning electron microscope (SEM; S-4500, Hitachi) equipped with an EDS spectrometer (Sigma, Kevex). SEM images of the CsMPs are shown in Fig. S6 and the radioactivity determined with imaging plates is appended to the images. The identified CsMPs were thinned to an X-ray and electron-transparent thickness using an FIB system with a micro-sampling unit (FB-2100, Hitachi).

### Glass synthesis

Glass with a similar composition to that of the CsMPs was synthesised as a reference material. The synthesis processes were the same as previously reported^10^ but we used a platinum instead of an alumina crucible and reduced the amount synthesised at one time for more rapid quenching. The target composition was 0.6K_2_O–0.6Rb_2_O–10.2Cs_2_O–10.9ZnO–7.7Fe_2_O_3_–3.3SnO_2_–66.7SiO_2_ (wt%), which is the averaged composition of the CsMPs reported in our previous study^10^ but without chlorine. The resulting glass was homogeneous and no phase separation was observed as shown in Fig. S7. A fragment around the centre of the crucible was crushed in an agate mortar and dispersed on carbon tape. The tape was carbon coated and the glass was thinned to an X-ray and electron-transparent thickness in the same manner as that of the CsMPs. After the glass was crushed and dissolved in HF solution, the composition of the synthetic glass was estimated using ICP analysis. The Cs atoms was measured by ICP-mass spectrometry (SCIEX-ELAN 6000, PerkinElmer), and the other atoms were determined by ICP-optical emission spectrometry (SPS3520, Hitachi)^57^.Fe-Mössbauer measurements were also performed for the crushed glass in a conventional transmission mode at room temperature on a Mössbauer spectrometer (Model-222, Topologic System) with a ^57^Co(Rh) source (925 MBq). Curve fitting of the Mössbauer spectra was performed by a nonlinear least-squares method using a MossWinn 4.0Pre program, assuming that all spectra were composed of Lorentzian-shaped peaks. The isomer shift and Doppler velocity scale were calibrated with respect to the sextet of α-Fe at room temperature.

### STXM

STXM analysis was conducted in transmission mode using a compact STXM system installed at BL-13A in the Photon Factory, KEK (Tsukuba, Japan)^33^. The thin samples were placed on a piezo-driven stage in a chamber purged with 0.1 atm of helium. The samples were scanned using an X-ray beam focused with a Fresnel zone plate. Mapping of transmitted X-ray from the pre- to post-edges of the interested elements was recorded using the image stacking method^34^. XANES spectra at each edge were obtained using the aXis2000 software. The Fe oxidation state maps were acquired using a SVD method in the software.

### (S)TEM

The thin specimens of the CsMPs were observed using a TEM (JEM-2010, JEOL) operated at 200 kV to characterise the inner structures of the CsMPs. EDS analysis was conducted using a STEM (JEM-2800, JEOL) operated at 200 kV equipped with a silicon drift detector (X-Max^N^ 100 TLE, Oxford Instruments). The probe size and current were ~1 nm and ~1 nA, respectively, and the collection angle (2*θ*) of the annular dark-field (ADF) detector was 46–208 mrad. Sample thickness measurement using EELS was also conducted using a spectrometer (Enfina, Gatan) attached to the STEM. The collection semi-angle was ca. 27 mrad and the full-width at half-maximum of the zero-loss peak was ca. 1.2 eV.

## Supplementary information

Supplementary Information.

## Data Availability

The data that support the findings of this study are available from the corresponding author upon reasonable request.
