# Supplementary material for: Reactor environment during the Fukushima nuclear accident inferred from radiocaesium-bearing microparticles
Source: Sci Rep. 2020 Jan 28;10:1352. doi: 10.1038/s41598-020-58464-y (PMC6987194; doi:10.1038/s41598-020-58464-y)
Supplement: Supplementary file 1 — Supplementary Information. [file 41598_2020_58464_MOESM1_ESM.pdf]

Supplementary Information

Reactor environment during the Fukushima nuclear accident inferred from radiocaesium-bearing microparticles

Taiga Okumura[1,*], Noriko Yamaguchi[2], Hiroki Suga[1], Yoshio Takahashi[1], Hiroyo Segawa[3], and Toshihiro Kogure[1]

[1]The University of Tokyo, Department of Earth and Planetary Science, Graduate School of Science, 7-3-1 Hongo, Bunkyo-ku, Tokyo 113-0033, Japan
[2]Institute for Agro-Environmental Sciences, NARO, 3-1-3 Kannondai, Tsukuba, Ibaraki 305-0864, Japan
[3]National Institute for Materials Science, 1-1 Namiki, Tsukuba, Ibaraki 305-0044, Japan

*Corresponding author E-mail: okumura@eps.s.u-tokyo.ac.jp

Determination of Na/Si ratio in CsMPs

To determine the atomic concentration in the specimens from EDS spectra, particularly for light elements, X-ray absorption inside the specimens needs to be corrected because their characteristic X-ray has a low energy and is considerably absorbed. First, the EDS spectra of the synthetic glass with a similar composition to that of the CsMPs but without Na were acquired from various thickness regions and the intensity ratio of the Zn L and Zn Kα peaks ($I_{ZnL}/I_{ZnK\alpha}$) was determined for each spectrum. Assuming that X-ray exponentially decays inside the materials and Zn Kα is hardly absorbed because of its high energy, the linear relationship between Ln($I_{ZnL}/I_{ZnK\alpha}$) and t/λ was obtained as shown in Fig. S3a, where t and λ are the specimen thickness and inelastic mean free path of the incident electrons in the specimen, respectively, and t/λ was determined by the electron energy-loss spectra (EELS) from each point of the specimen. The descending regression line corresponds to the absorption of Zn L with a low energy. Using this relationship, I_{ZnL} of the spectra from CsMP-HD and CsMP-Ma was estimated from their $I_{ZnK\alpha}$ and t/λ, and the Na Kα peak intensity ($I_{NaK\alpha}$) was obtained by subtracting I_{ZnL} from the overlapped peaks of Zn L and Na Kα. Here, we assume that λ for the synthetic glass and CsMPs is the same because λ is approximately a function of the density and mean atomic number of the specimen.[1,2] Furthermore, the decay rates inside the materials for Zn L and Na Kα are the same because their energy is nearly identical. Accordingly, the Na Kα peak intensity without absorption ($(I_{NaK\alpha})_{t=0}$) was obtained using the relationship shown in Fig. S3a. The absorption of Si Kα corresponding to specimen thickness can be estimated in the same manner as previously described and the linear relationship between Ln($I_{SiK\alpha}/I_{ZnK\alpha}$) and t/λ for the synthetic glass was also obtained, as shown in Fig. S3b. Using this relationship, we calculated the Si Kα peak intensity without absorption ($(I_{SiK\alpha})_{t=0}$) for CsMP-HD and CsMP-Ma. Using the intensity ratio ($(I_{NaK\alpha}/I_{SiK\alpha})_{t=0}$) of Na Kα and Si Kα without absorption, as previously determined, and the k-factor converted from the mass ratio to the atomic ratio ($k_{NaSi} = 1.373$) for the microscope, the Na/Si atomic ratio for CsMP-HD and CsMP-Ma was determined (Table 1).

References

1. Malis, T., Cheng, S. C. & Egerton, R. F. EELS log-ratio technique for specimen-thickness measurement in the TEM. *J. Electron Microsc. Tech.* **8**, 193–200 (1988).
2. Iakoubovskii, K., Mitsuishi, K., Nakayama, Y. & Furuya, K. Thickness measurements with electron energy loss spectroscopy. *Microsc. Res. Tech.* **71**, 626–631 (2008).

Table S1. Composition of synthetic glass other than oxygen

SiO_2	K_2O	Fe_2O_3	ZnO	Rb_2O	SnO_2	Cs_2O	Total	
70.2	0.5	8.0	9.4	0.5	2.3	9.1	100.0	(wt%)

Table S2. Calculated Mössbauer parameters for the bulk synthetic glass

	Isomer shift (mm/s)	Quadrupole splitting (mm/s)	FWHM (mm/s)	Relative area (%)
Fe^{2+}	0.886(8)	2.20(1)	0.74(2)	37(1)
Fe^{3+}	0.401(7)	0.77(1)	0.93(3)	63(1)

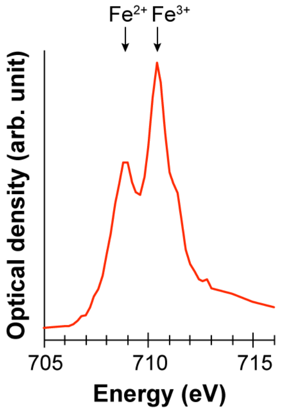

Figure S1. XANES spectrum of the synthetic glass.

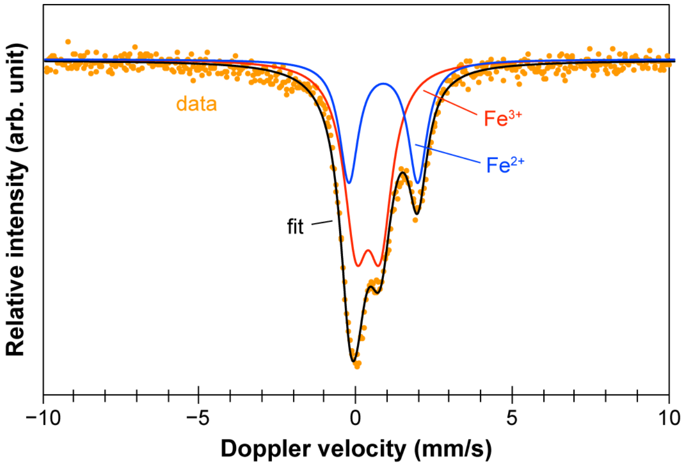

Figure S2. Mössbauer spectrum of the bulk synthetic glass.

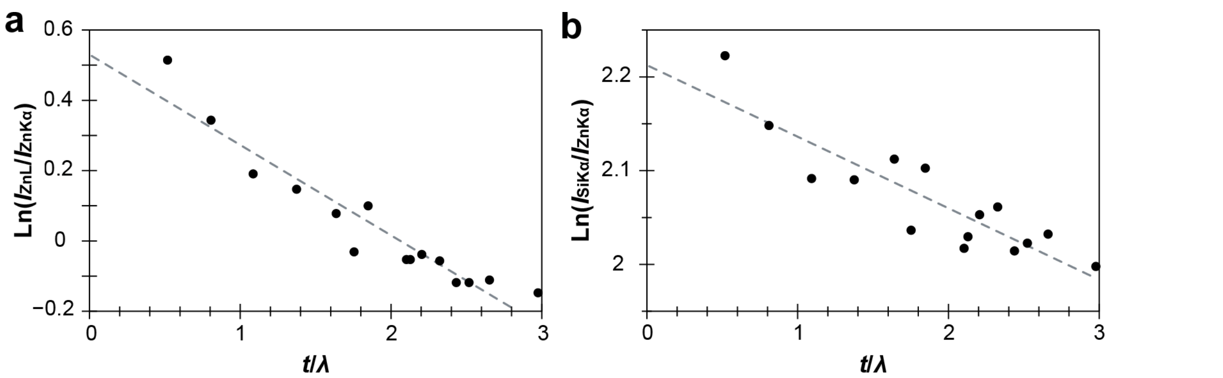

Figure S3. (a) Relationship between Ln(I_{ZnL}/$I_{ZnK\alpha}$) and t/λ for synthetic glass. (b) Relationship between Ln($I_{SiK\alpha}$/$I_{ZnK\alpha}$) and t/λ for synthetic glass.

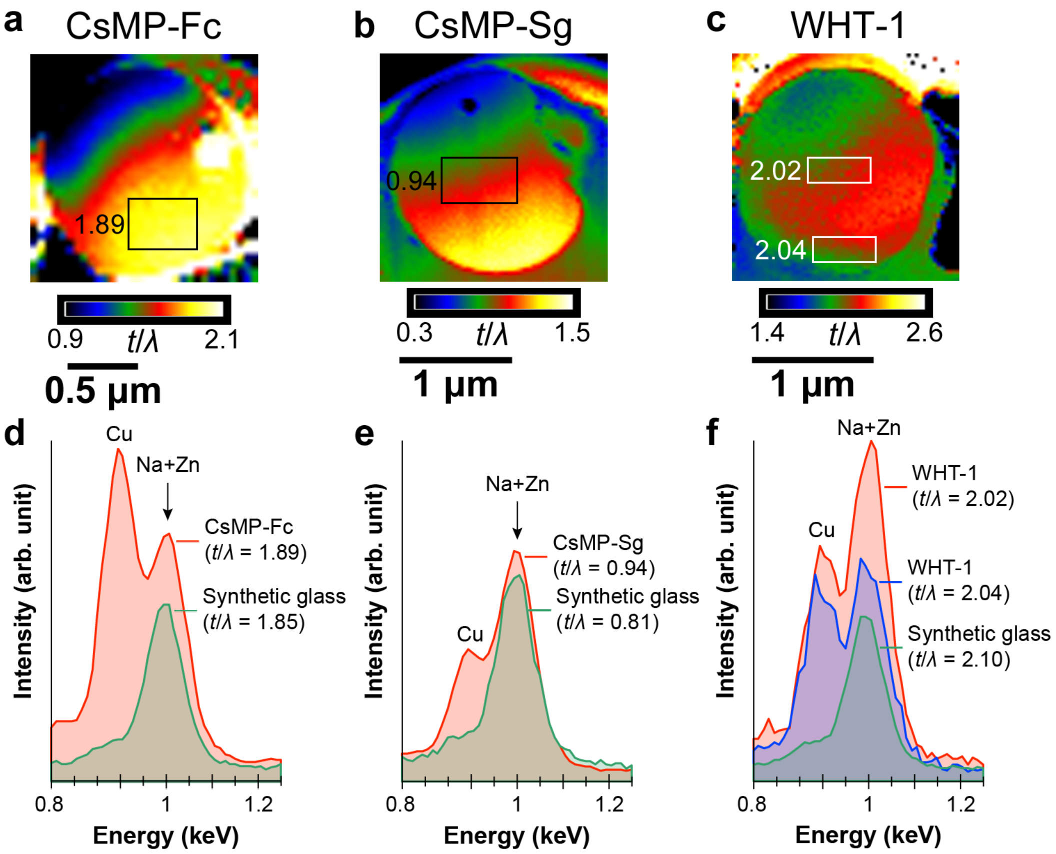

Figure S4. Relative thickness maps of CsMP-Fc (a), CsMP-Sg (b), and WHT-1 (c). Rectangles show the location where the EDS spectra were obtained. The values next to the rectangles indicate the relative thickness of each rectangular area. (d–f) EDS spectra obtained from the rectangles in (a–c) at the Na Kα and Zn L peaks. Spectra obtained from the synthetic glass with similar relative thickness are also shown. The appearance of Cu peaks at 0.9 keV in this figure, which do not appear in Fig. 5, is due to different types of cupper substrates supporting the specimen.

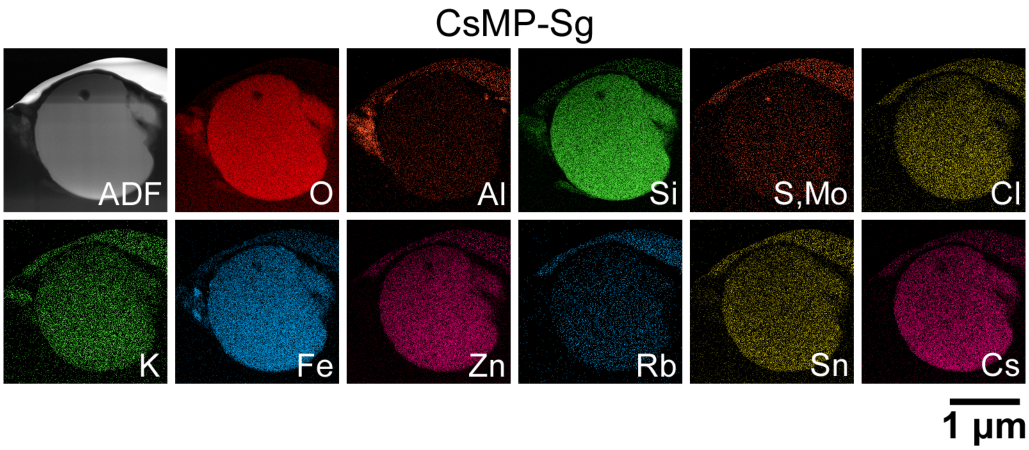

Figure S5. ADF image and corresponding elemental maps of CsMP-Sg obtained using STEM-EDS.

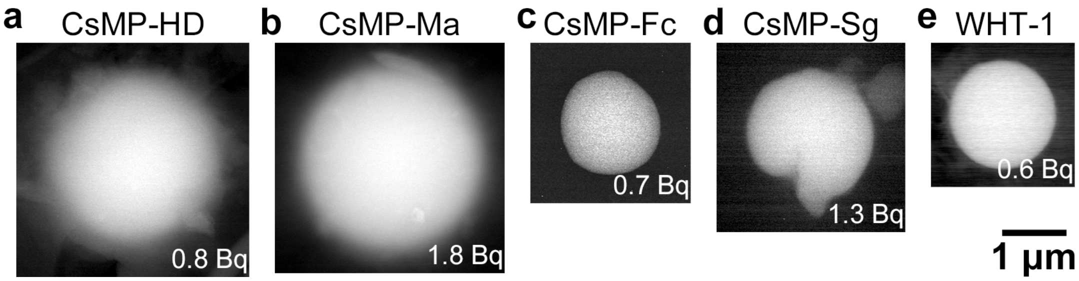

Figure S6. SEM images of the CsMPs. The images were taken using back-scattered electrons at an accelerating voltage of 20 kV. The radioactivity determined with imaging plates is appended to the images.

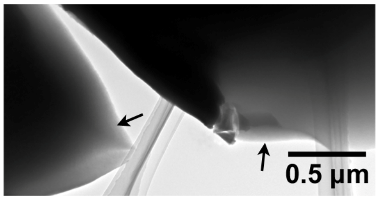

Figure S7. TEM image of synthetic glass. Arrows indicate glass fragments.
